# Differentiation between Cerebral Hemorrhage and Contrast Extravasation Using Dual Energy Computed Tomography after Intra-Arterial Neuro Interventional Procedures

**DOI:** 10.5334/jbsr.2083

**Published:** 2020-11-25

**Authors:** Yasmine Zaouak, Niloufar Sadeghi, Nicolae Sarbu, Noémie Ligot, Boris Lubicz

**Affiliations:** 1 Erasme Hospital, BE

**Keywords:** dual energy ct, cerebral haemorrhage, blood brain barrier, contrast extravasation, neuro interventional

## Abstract

**Purpose:**

To evaluate the value of dual-energy computed tomography (DECT) in differentiating cerebral hemorrhage from blood brain barrier (BBB) disruption after neuro-interventional procedures with intra-arterial injection of iodinated contrast material.

**Material and methods:**

This prospective study was approved by the local ethics committee, and informed consent was obtained for all patients. Thirty five patients with acute ischemic stroke or un-ruptured brain aneurysm who had received intra-arterial administration of iodinated contrast material were evaluated using DECT at 80 and 150 kV immediately after the procedure.

A three-material decomposition algorithm was used to obtain virtual non-contrast (VNC) images and iodine overlay maps (IOM). A follow-up examination (brain magnetic resonance imaging MRI or conventional CT) was used as the standard of reference for hemorrhage, defined as a persistant hyperdensity on a conventional CT or T2* hypo-intensity on brain MRI. The diagnostic values of DECT in differentiating hemorrhage and iodinated contrast material were obtained.

**Results:**

Mixed images obtained with DECT showed intra-parenchymal or subarachnoid hyperattenuation in 18/35 patients. Among these, 16 were classified (according to VNC images and IOM) as contrast extravasations and two with a mixture of hemorrhage and contrast material. On follow-up imaging, there were two patients with hemorrhage. The sensitivity, specificity, and accuracy of DECT in the identifying hemorrhage was calculated as 67% (2/3), 100% (32/32) and 97% (32/33) respectively.

**Conclusion:**

DECT allows an early and accurate differentiation between cerebral hemorrhage and BBB disruption after intra-arterial neuro-interventional procedures.

## Introduction

Neuro-interventional procedures are currently being widely developed, for various indications. The intra-arterial approach is now the first line treatment in cases of both ruptured and un-ruptured cerebral aneurysms [1]. In case of acute ischemic stroke, thrombectomy has significant clinical advantages for patients as compared to systemic treatment alone [2], with platelet anticoagulant/antiaggregant treatment being administered in both groups of patients to avoid vascular embolism [2,3,4]. However, there is a risk of intracerebral hemorrhage (ICH) following neuro-interventional procedures, thus potentially modifying the therapeutic decisions [5]. The frequency of cerebral hemorrhage can reach 15.5% in the first 24 to 36 hours following intra-arterial revascularization in case of acute ischemic stroke [6]. In case of intra vascular treatment of un-ruptured intracranial aneurysms, the rate of per-operative perforation varies between 0 and 1.3% [3].

Brain Computed Tomography (CT) right after a neuro-interventional procedure may show hyperattenuating areas with a frequency varying across studies between 16% and 85% after intra-arterial revascularization by thrombolysis [8,9,10] and/or thrombectomy [4,7,8,10,11,12]. They are found in 20 to 50% of patients after intravascular treatment of cerebral aneurysms [3,13,14]. They represent either ICH or blood brain barrier (BBB) rupture with contrast extravasation [3,4,7]. Their complete resolution on follow-up brain imaging, occasionally with no brain hemorrhage, can be explained by the phenomenon of temporary extravasation of the contrast medium as a result of ruptured BBB [3,4,5,7,8,9,10,11,13,14]. The density of the contrast medium and that of the hemorrhagic areas being close to one another on conventional brain CT, it may be difficult to distinguish between brain hemorrhage and extravasation of contrast medium straight after a neuro-interventional procedure [5,8,15]. Extravasation of contrast agent, as some studies have suggested, can be considered as a good prognostic sign and by extension should not always be misinterpreted as hemorrhage alone [5]. It is therefore necessary to perform a 24h follow-up exam, thus delaying clinical decisions and exposing the patient to an additional dose of radiation. Early detection of intraparenchymal hemorrhage can lead to adjust platelet anticoagulant/antiaggregant treatment and potentially limit hemorrhage growth. Resorting to early cranial magnetic resonance imaging (MRI) would be justified in this case [16]. However, access to this imaging technique may limited in most centers.

DECT is based on the use of X-ray beams of different energy levels (usually 80 kilovolts and 140 kilovolts) allowing the separation of different materials spectra that have different attenuation properties upon exposed to different X-ray energies [15]. DECT has been previously used to differentiate between hemorrhagic lesion and iodinated contrast material staining [17,18]. Prior studies have also reported the use of this technique in patients treated by intra-arterial thrombolysis [19,20,21,22,23] or thrombectomy [22,23], but to the best of our knowledge, none has evaluated patients treated by endovascular route for unruptured brain aneurysms.

The purpose of this study was to evaluate DECT for the differential diagnosis between brain hemorrhage and BBB rupture after neuro-interventional procedures with intra-arterial contrast injection in patients with acute stroke or un-ruptured brain aneurysm.

## Materials and Methods

### Patients

This prospective study was approved by the local ethics committee, and informed consent was obtained from all patients. From, December 2015 to April 2016 we collected consecutive patients who underwent intra-arterial neuro-interventional procedures either for thrombectomy in case of stroke or for closure of un-ruptured aneurysms. In all patients DECT was performed within 60 minutes of the procedure. Another 24-48h follow-up exam (brain MRI or conventional brain CT) was also obtained [24,25].

### Therapeutic approach

Patients treated for un-ruptured brain aneurysm with stent and coils – or flow diverter stent received 300mg of acetylsalicylic acid and 240 mg of clopidogrel the day before as well as the day of the procedure.

All patients received heparin-based anticoagulant treatment during the intervention (initial intravenous bolus of 70 units/kg then continuous infusion of 35units/kg/h).

### Imaging acquisition

All DECT were performed using a third generation scanner (Somatom Force®, Siemens Healthcare, Forchheim, Germany). References of tubes A and B are as follows: Tube A: 80kV, and 310 mAs; Tube B: 150 kV, 207 mAs, using a pewter filter. Collimation was set to 64 × 0,6 mm. The system used a dose modulation (Care Dose4D®, Siemens). Rotation time and pitch were respectively 1.0 seconds and 0.7.

MRI were performed on either 1.5T or 3T scanners (Intera 1.5T®, Achieva 3T®, Ingenia 3T®; Philips Medical Systems, Best, Netherlands) depending on availability. The following sequences were used: axial planes T1-weighted (Echo Time (TE) = 12ms, Repetition Time (TR) = 647ms; or TE = 2.46ms, TR = 230ms), axial T2-weighted (TE = 100ms, TR = 6514ms; or TE = 80ms, TR = 6700ms), axial diffusion weighted imaging(TE = 68ms, TR = 3592ms; or TE = 59ms, TR = 3230ms), axial T2 gradient echo (TE = 21ms, TR = 1056ms; or TE = 18ms, TR = 899ms), and axial fluid attenuation inversion recovery (FLAIR) (TE = 120ms, TR = 6500ms, Inversion Time (TI) = 2100ms; or TE = 91ms, TR = 9000ms, TI = 2500ms). When MRI was unavailable or in case of contraindications to its use (pacemaker, claustrophobia), we performed a conventional brain CT for follow-up. Conventional CT was performed on a 16-slice scanner (Emotion 16®, Siemens Healthcare, Forchheim, Germany). The reference of the tube was 110 kV and 220 mAs. Collimation was 16 × 0.6 mm. Rotation time and pitch were respectively 0.6 seconds and 0.55. We chose a 24-48h time lapse (as per hospital clinical routine) between DECT and the next follow-up exam.

### Image reconstruction

Virtual Non-Contrast (VNC) imaging and iodine overlay maps (IOM) were recreated on a multi-modality workstation (SyngoVPCT-Neuro; Siemens Healthcare, Forchheim, Germany) thanks to an algorithm for material decomposition. Mixed images were created by merging data acquired at 80 and 150 kV (50% of data at 80kV and 50% of data at 150kV, arbitrarily). VNC images were obtained by subtracting the iodine absorption spectra from each voxel.

### Image analysis

On DECT, we analyzed the absence or presence of hyperattenuation on the mixed images. We then classified these hyperattenuations based on the VNC and IOM images as follows: 1) Brain hemorrhage if hyperattenuation was not found on IOM; 2) BBB rupture if hyperattenuation only existed on IOM, or 3) Brain hemorrhage and BBB rupture if it existed on both VNC and IOM images (Table 1).

**Table 1:** Different patterns of hyperattenuation on virtual non contrast (VNC) images and iodine overlay map (IOM).

Diagnostic	VNC images	IOM

Brain hemorrhage	+	–
Blood brain barrier rupture	–	+
Brain hemorrhage and blood brain barrier rupture	+	+

*Note:* + = hyperattenuation present; – = hyperattenuation absent.

Follow-up exams were considered positive for the diagnosis of a brain hemorrhage if signs of hemorrhagic transformation were found in the hyperattenuating areas detected on DECT. All images were analyzed by two radiologists (N.S.M.; 16 years of experience in Neuroradiology; Reader 1 and N.S.; 3 years of experience in Neuroradiology; Reader 2) and by a last year medical student (Y.Z.; Reader 3). All reconstructed images (VNC and IOM) were anonymized and ordered by date of test, which was unknown to the readers. Each reader had two to four reading sessions. For the Reader 3, the reading sessions were preceded by three training sessions. For each patient, the reading of images followed a specific order: first mixed images followed by VNC images, IOM and follow-up examinations. A consensus was also obtained between Readers 1 and 2 for all data.

### Radiation dose

We took note of the volumetric CT dose index (CTDI vol) and the dose length product (DLP) for each examination CT (DECT and conventional CT).

### Statistical analysis

We used statistical software for biomedical research (MedCalc®, version 10.0, Mariakerke, Belgium). Cohen›s kappa test was used to estimate inter-rater agreement. We then calculated sensitivity and specificity, positive predictive value, negative predictive value as well as the accuracy of DECT in diagnosing cerebral hemorrhage.

## Results

We consecutively collected 35 patients (29 females and 6 males; mean age = 55 years; range = 44–66 years). Six patients were treated for stroke by intra-arterial thrombectomy and 29 were treated for un-ruptured aneurysms by intravascular treatment.

As shown on Table 2, the concordance between the readers to characterize CT hyperattenuation was very good to excellent (Kappa coefficient ranges from 0.89 to 1).

**Table 2:** The inter-observer concordance kappa coefficients obtained between the readers in characterizing hyperattenuatting abnormalities on cross-sectional imaging post neurointerventional procedures.

	Reader 1/Reader 2	Reader 1/Reader 3	Reader 2/Reader 3

Mixed images	0.89	0.89	1
VNC images	1	1	1
IOM	1	1	1
FU MRI	1	1	1
FU CT	1	1	1

After consensus reading, hyperattenuations were found in 18 patients out of 35 patients (51%); 5 out of 6 patients (83%) with thrombectomy and 13 out of 29 patients (45%) with intravascular treatment for an aneurysm.

Table 3 summarizes the percentages of extravasation of contrast medium and/or cerebral hemorrhage as defined by the readers using the different DECT maps.

**Table 3:** Frequency of extravasation of contrast medium and brain hemorrhage on DECT according to all readers.

	All patients (N = 35)	Thrombectomy (N = 6)	Aneurysm (N = 29)

Extravasation of contrast medium without ICH	16/35 (45%)	4/6 (67%)	12/29 (41%)
ICH	–	–	–
Extravasation of contrast medium and ICH	2/35 (5.7%)	1/6 (17%)	1/29 (3%)

Of the 35 follow-up examinations (26 MRI, 9 conventional CT), three (2 MRI, 1 conventional CT) were positive for cerebral hemorrhage. Table 4 summarizes the true and false positives as well as the true and false negatives of DECT for post-procedural brain hemorrhage.

**Table 4:** Criteria for true positive, false positive, true negative and false negative for hemorrhage on DECT and follow-up (FU) imaging.

	Hyper attenuation on DECT			Findings at FU	

Diagnosis	*VNC images*	*IOM*	*Conventional CT*	*Brain MRI*

True positive (N = 2)	+	+/–	Persistant hyperattenuation	Brain hemorrhage
False positive (N = 0)	+	+/–	Washout	No brain hemorrhage
True negative (N = 32)	–	+/–	Washout	No brain hemorrhage
False negative (N = 1)	–	+/–	Persistant hyperattenuation	Brain hemorrhage

*Note:* + = hyperattenuation present; – = hyperattenuation absent; +/– = hyperattenuation present or absent.

**Figure 1:**
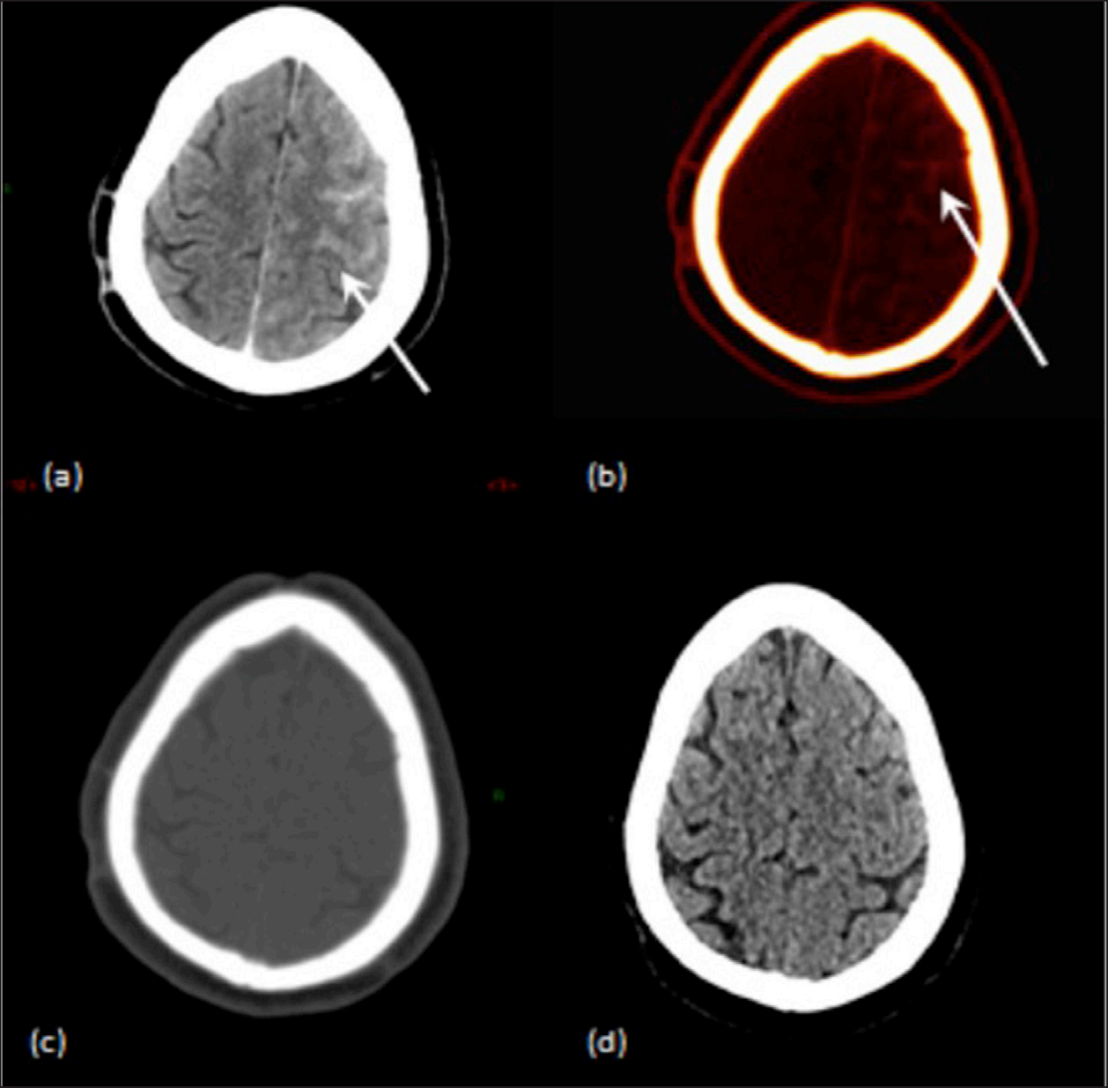
A case of a 68 year-old patient who underwent intravascular treatment of an unruptured brain aneurysm of the anterior communicant artery. **(a)** Mixed DECT image shows a left hemispheric hyperattenuation in the subarachnoid space. **(b)** IOM shows contrast material staining in the same area. **(c)** VNC image shows no hyperattenuation thus rule out hemorrhage. **(d)** Follow-up conventional CT shows no sign of brain hemorrhage.

**Figure 2:**
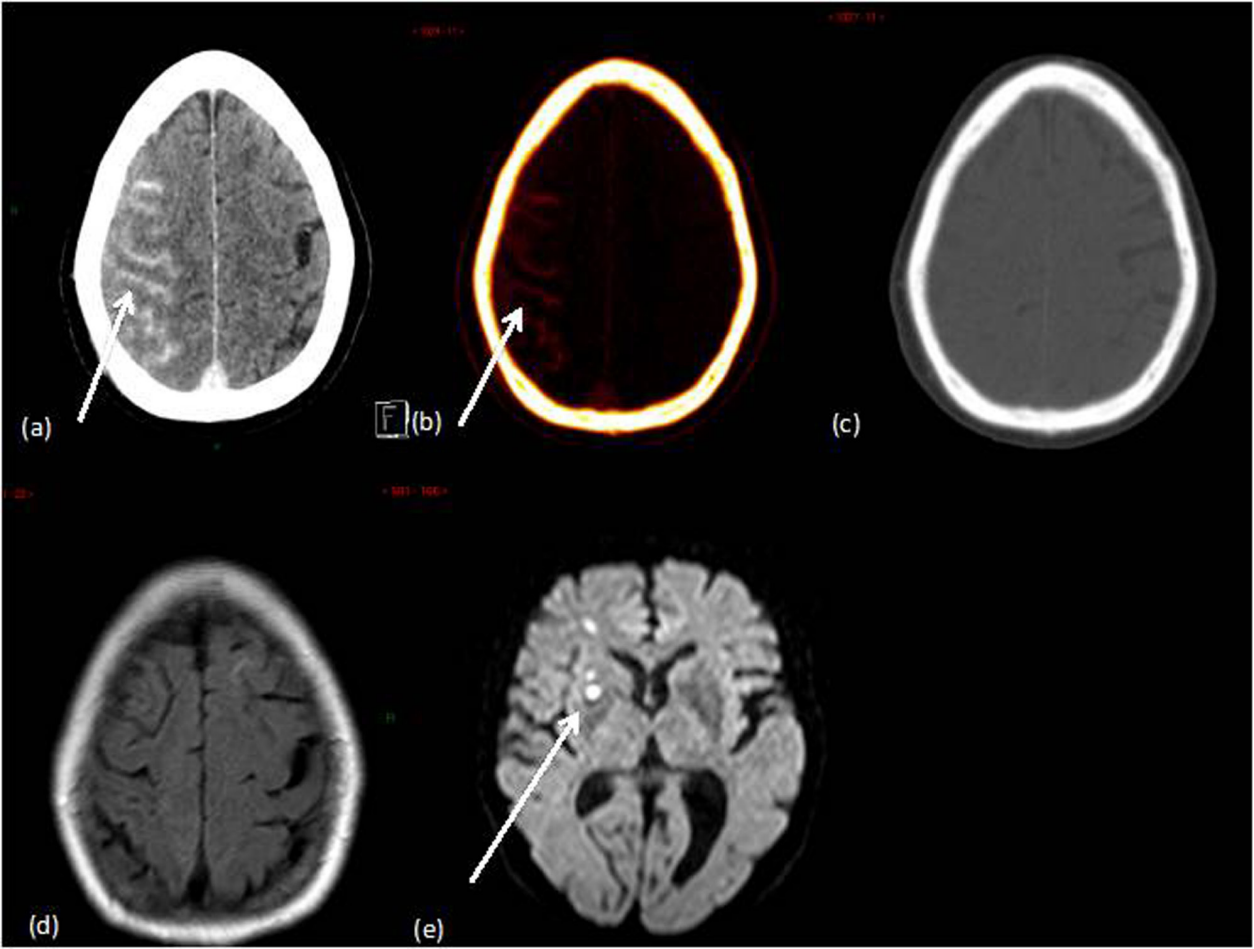
The case of a 69 years old patient who underwent intra-arterial treatment of an un-ruptured cavernous carotid aneurysm. DECT was performed immediately after the procedure. **(a)** Mixed DECT image shows a right hemispheric hyperattenuation in the subarachnoid space. **(b)** IOM shows contrast material staining in the same area. **(c)** VNC image shows no hyperattenuation thus rule out hemorrhage Twenty-four hours after the procedure, the patient progressively developed confusion and left brachiofacial hemiparesis. Follow-up MRI was performed and showed ischemic lesions of embolic appearance in the right hemisphere. **(d)** Axial T2 fluid attenuation inversion recovery sequence (brain MRI) shows no signs of brain hemorrhage. **(e)** Diffusion weighted imaging shows ischemic lesions of embolic appearance in the right hemisphere.

Sixteen patients demonstrated BBB rupture without brain hemorrhage and thus were among the true negative patients (Figures 1 and 2).

**Figure 3:**
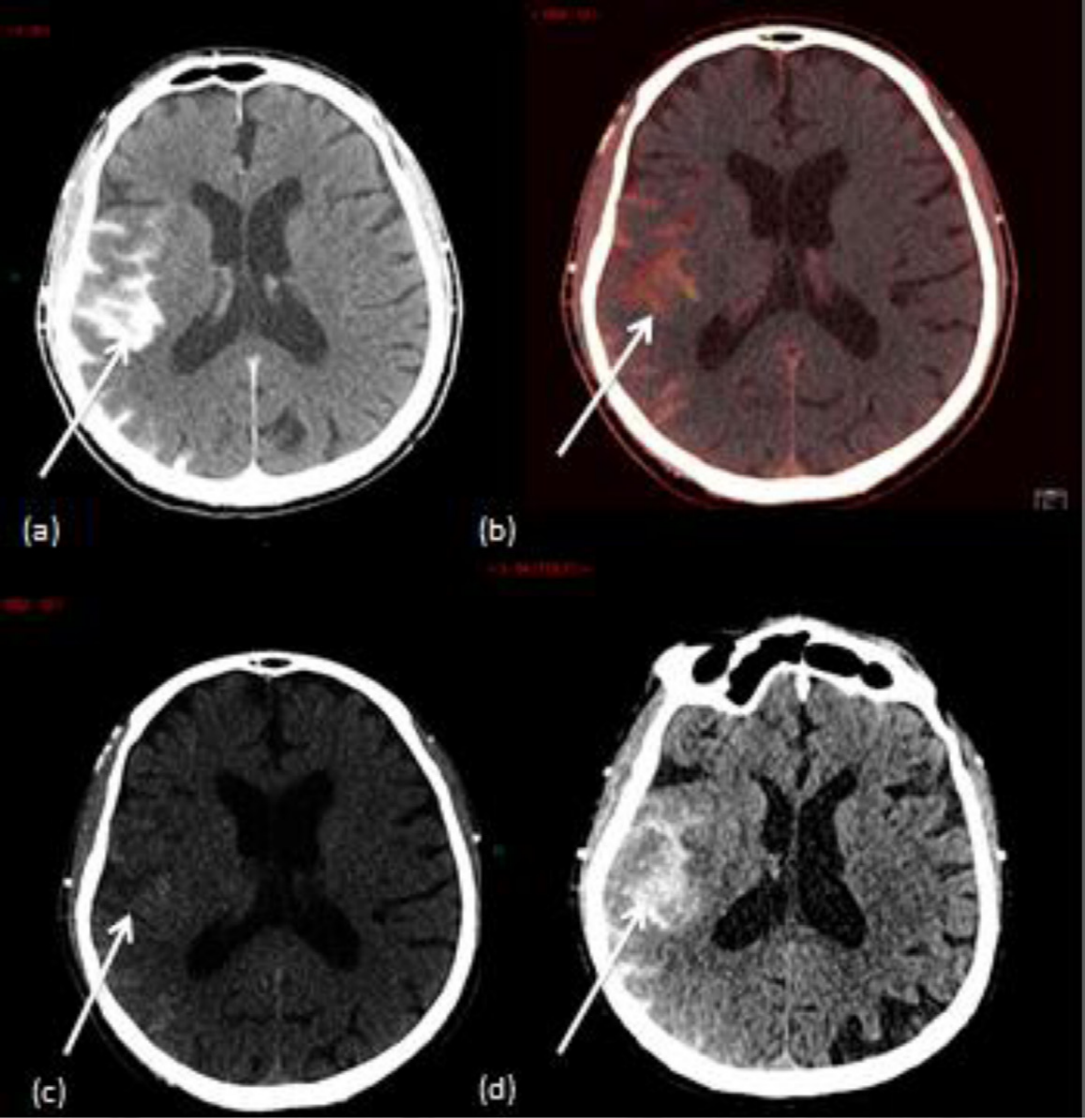
A case of a 77 years old patient who underwent intra-arterial thrombectomy (right middle cerebral artery). **(a)** Mixed DECT image shows an hyperattenuation in the perisylvian cortex. **(b)** IOM shows contrast material staining in the perisylvian cortex. **(c)** VNC image shows a subtle hyperattenuation in the perisylvian cortex. **(d)** Follow-up conventional brain CT shows a persistant hyperattenuation in the perisylvian cortex which confirms brain hemorrhage.

One patient showed brain hemorrhage that was confirmed by follow-up conventional brain CT (Figure 3). There were two true positive patients.

**Figure 4:**
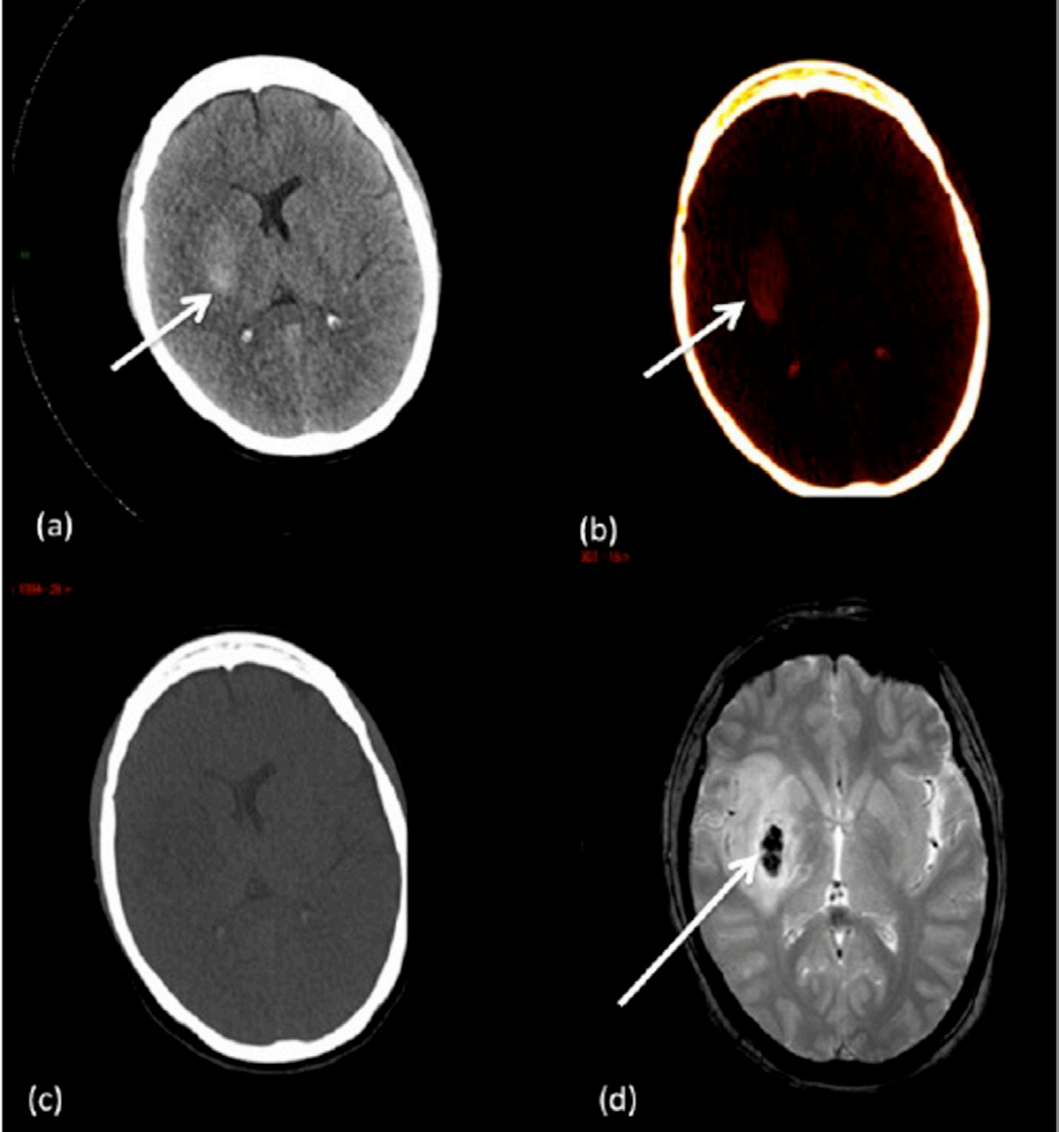
A case of an 45 years old patient who underwent intra-arterial thrombectomy (right middle cerebral artery). **(a)** Mixed DECT image shows an area of hyperattenuation in the right lenticular nucleus. **(b)** IOM shows contrast material staining in the right lenticular nucleus. **(c)** VNC image shows no hyperattenuation thus rule out hemorrhage. **(d)** Follow-up brain MRI shows hemorrhagic transformation of the ischemic lesion in the right lenticular nucleus.

One patient showed BBB rupture without brain hemorrhage, but follow up examination showed hemorrhagic transformation of the ischemic lesion (Figure 4). This patient was the only false negative.

The sensitivity and specificity of DECT in the identification of brain hemorrhage were 67% (2/3) and 100% (32/32), respectively. Positive predictive value, negative predictive value and accuracy were 100% (2/2), 97% (32/33) and 97% (32/33), respectively.

The mean total effective dose of irradiation of DECT (21.72 mGy) was comparable to that of a conventional brain CT in our study (26.96 mGy) (Table 5).

**Table 5:** Mean Volumetric Computed Tomography Dose Index (CTDI vol), average Dose Length Product (DLP) and mean total effective dose of irradiation in patients who underwent DECT and conventional brain CT.

	DECT	Conventional brain CT

Mean CTDI vol (mGy)	21.72	26.96
Average DLP (mGy.cm)	400	470
Mean total effective dose of irradiation	0.84	0.98

## Discussion

In our series of patients, DECT performed after intra-arterial neuro-interventional procedure can be used to diagnose a cerebral hemorrhage with excellent specificity and relatively good sensitivity (considering the small number of true positive patients).

Several previous studies have evaluated the DECT in diagnosing brain hemorrhage after an endovascular treatment by thrombolysis and/or thrombectomy.

The study of Gupta et al. [19] showed that the DECT has a sensitivity of 100%, a specificity of 91% and an accuracy of 93% for the diagnosis of cerebral hemorrhage. Phan et al. [20] studied 40 patients who were injected with contrast medium intravenously or intra-arterially. Among these patients, sensitivity and specificity of the DECT for the same diagnosis were 100% and 84% respectively. The studies led by Tijssen et al. [23] and Morhard et al. [22] showed similar results. It is important however to keep in mind that these studies differ in the way of injection of the contrast medium (intravenous or intra-arterial) and the treatment carried out (intra-arterial thrombolysis or thrombectomy).

Among our patients, there was one false negative for the diagnosis of cerebral hemorrhage by DECT, who also presented with a substantial BBB rupture after thrombectomy. We hypothesized that the hemorrhagic transformation within the ischemic lesion appeared after the DECT was performed. It was probably facilitated by the BBB rupture. This would explain why it didn’t appear on VNC images of the DECT. This phenomenon of hemorrhagic transformation of BBB rupture has previously been described [8,23]. In addition, BBB rupture following an acute ischemic stroke has been associated with an elevated risk of subsequent hemorrhagic transformation and a poor clinical prognosis [9,21,26].

One patient showed brain hemorrhage that was confirmed by follow-up conventional brain CT. When brain hemorrhage was discovered, anticoagulation therapy (which was its chronic treatment for an atrial fibrillation) was stopped during two days. This example illustrates the benefice of early detection of intraparenchymal hemorrhage in order to adjust therapy [6].

We have not observed a false positive case in the diagnosis of cerebral hemorrhage with the DECT. This type of presentation has, however, previously been reported. The intra-parenchymatous hyperattenuations described were actually zones of mineralization related to parenchymatous calcifications [19,20] and in one case, due to a metallic artifact [20]. The calcifications as well as the metallic artifact coexisted on the VNC image and on the IOM [19,20]. Furthermore, it has been proven that the presence of a fourth type of material (besides brain tissue, iodine and blood) is a limiting factor for the software for decomposition of three types of material [15].

We have not encountered any cases of patients presenting an isolated cerebral hemorrhage, without a concomitant BBB rupture. This seemed obvious, since hemorrhage following a neuro-interventional procedure is always associated with a breach of a vascular wall, and would therefore be combined with an extravasation of contrast medium [7,21]. Yet, studies led by Gupta et al. [19] and by Phan et al. [20] described intra-parenchymatous hyperattenuations corresponding to cerebral hemorrhage with no extravasation of contrast medium. We do not have a clear explanation for this finding. However it is important to note that patients enrolled in these studies were intravenously injected with contrast medium after being given a brain CT. This test was done for the assessment of tumor lesions or trauma. In contrast, all our patients received a DECT immediately after the intra-arterial procedure. As a consequence, our results could be compared with those of other studies that set up the same method of use of the DECT as we did.

Lastly, our study showed the total effective radiation dose of a DECT was comparable to that of a conventional brain CT. This result is in accordance with what has been reported in the literature [19,20,23]. Knowing that using the conventional brain CT in this case requires a follow-up exam, it would seem wise to promote the use of DECT. The early use of brain MRI would thus be justified in this case [16]. However, access to MRI may be limited.

Our study was limited in certain aspects. First, our patient population possessed certain heterogeneity with a limited number of patients treated by thrombectomy. Secondly, there were only three cases with post-procedural brain hemorrhage, limiting the diagnostic values observed for the diagnosis of a brain hemorrhage on DECT. This might have been caused by the facts that reference examinations were carried out 24-48 hours after the procedure and that MRI which is more sensitive than CT for hemorrhage was not done in every patient by the time of DECT because of organizational constraints. Further studies designed to overcome the limitations of the current work, especially in terms of patient number and design will be needed to confirm or findings.

In conclusion, our study shows that the DECT is an efficient tool for early differential between a cerebral hemorrhage and BBB rupture following intra-arterial neuro-interventional procedures.
